# Psychological trauma of Middle East Respiratory Syndrome victims and bereaved families

**DOI:** 10.4178/epih.e2016054

**Published:** 2016-12-02

**Authors:** Minyoung Sim

**Affiliations:** Department of Stress and Anxiety Disorder, National Center for Mental Health, Seoul, Korea

An epidemic is a very special type of disaster that is accompanied by social stigma and discrimination. Historically, when you look at the period of an epidemic, it is easy to see how many people have tried to control their anxiety by persecuting and discriminating against minorities. During the Black Death epidemic, Gypsies and Jews were persecuted due to rumors that they were poisoning water. There also was a period of fear and persecution of lepers due to rumors that they were harming their children. This is tragic for victims of infectious disease as they have difficulties receiving social support despite being direct victims themselves.

In the early days of the Sewol ferry disaster, the social environment was full of support for the bereaved families, whereas the general public was busy avoiding the evacuees and their families in the early period of Middle East Respiratory Syndrome (MERS).

One family affected with MERS said, “Everyone avoided us after hearing about a MERS patient in our family. Some shopkeepers threw things at us and shouted at us to leave.” Even after being treated and cleared from disease, many patients suffered from rumors and discrimination.

Some of the stories on the Internet, such as “the incubation period is longer than 14 days” and “there are asymptomatic patients who are infected,” branded these patients as potential risk factors for spread of infection. The experience of cold treatment by close relatives and neighbors gave these patients a deep scar. Once the MERS epidemic comes to an end, we should consider how rumors were inflated and how they affected us and our neighbors. This reflection is necessary because epidemics of infectious diseases will continue to recur in the future. To respond in a more mature way, we need to recognize that the rumors we have too easily accepted resulted in irreversible scars to primary victims by the disease and worsening public anxiety and fear. Underlying the fear and anxiety that prevailed during the MERS epidemic was the uncertainty that I, my family, or someone close to me could become infected. When uncertainties are high, rumors can be exaggerated.

Even if all questions for infectious diseases cannot be answered, uncertainties can be largely eliminated through efficient disaster management systems established by a group of experts. From a perspective of controlling disasters, it has tendency to delay or postpone providing information because of the belief that bad news will cause people to panic. However, it is important to provide accurate information about the disaster as soon as possible. Missing the appropriate time point could cause rapid spread of rumors. Thus, the ability to communicate with the public via a group of experts is crucial to reduce uncertainty among the general public.

