# Supplementary material for: Psychological trauma of Middle East Respiratory Syndrome victims and bereaved families
Source: Epidemiol Health. 2016 Dec 2;38:e2016054. doi: 10.4178/epih.e2016054 (PMC5177804; doi:10.4178/epih.e2016054)
Supplement: Supplementary file 1 [file epih-38-e2016054-supplementary.pdf]

-Commentary-

메르스 감염자 및 유가족의 심리적 트라우마

심민영

국립정신건강센터

전염병은 낙인과 차별을 동반하는 매우 특수한 형태의 재난입니다. 역사적으로 전염병이 유행했던 시기를 살펴보면 많은 사람들이 소수자를 박해하고 차별함으로써 자신의 불안을 다스리려고 했던 시도를 쉽게 찾아볼 수 있습니다. 흑사병이 유행하던 중세시기에는 집시와 유대인이 물에 독을 탔다는 소문을 믿고 이들을 학살했고, 근대에 와서도 나병환자들이 아이를 해친다는 루머 때문에 이들을 두려워하고 박해하던 시기가 있었습니다. 이처럼 직접적인 피해자임에도 불구하고 사회적인 지지를 받기 어렵다는 점이 감염병 피해자들의 비극이라고 생각합니다. 세월호 사고 초반에는 유가족에 대한 전폭적인 응원과 지지라는 사회적 분위기가 형성됐었던 반면에, 메르스 때는 초반부터 감염자와 그 가족들을 기피하기 바빴습니다. 어떤 유가족은 "우리 집에 메르스 환자가 발생했다는 것에 대해서 모두가 수근대고 우리를 피했다. 어떤 가게 주인은 물건을 던지듯이 주고는 어서 가라고 소리를 지르기도 했다." 라고 말하기도 했습니다. 완치판정을 받은 후에도 루머와 차별에 시달리며 위축된 생활을 하는 분들이 많았습니다. 인터넷에 떠도는 얘기들, 이를테면 '잠복기가 14일보다 길다고 하더라', '무증상 감염자가 있다더라' 와 같은 내용들은 그분들을 여전히 잠재적인 위험인으로 낙인찍는 대표적인 소문이었습니다. 가까운 친척과 이웃들로부터 냉대받은 경험은 그 분들에게 깊은 상처가 되었습니다. 사태가 종결되고 나면 메르스와 관련된 루머들이 어떻게 과장되어 퍼져나갔고, 결국 우리 자신과 이웃에 어떠한 영향을 미쳤는지 돌아볼 필요가 있다는 생각을 하게 됐습니다. 왜냐하면, 감염병은 앞으로도 계속 되풀이될 것이기 때문입니다. 메르스 때보다 더 성숙한 방식으로 대응하기 위해서는 우리가 너무나 쉽게 받아들였던 루머들이 당사자들에게는 씻을 수 없는 상처가 될 수 있다는 것, 그리고 그것이 오히려 불안과 공포를 확대시켰다는 점을 인식하고 반성할 필요가 있습니다. 메르스 사태 때 만연했던 공포나 불안의 기저에는 나와 내 가족, 나와 가까운 사람이 잘못될 수도 있다는 불확실성이 깔려있었습니다. 불확실성이 클 때 루머도 더 양산되는 특징이 있습니다. 전염병과 관련된 불안과 걱정에 전부 답할 수는 없더라도 전문가 집단의 역량과 효율적인 재난관리체계를 통해서 불확실성을 상당부분 타개할 수 있습니다. 재난을 수습하는 입장에서는 재난과 관련된 나쁜 소식이 사람들을 더 공황에 빠뜨릴 것이라고 생각해서 이러한 상황을 축소하거나 공표를 미루기 쉬운데, 위기 상황일 수록 파악된 정보를 최대한 솔직하게, 너무 늦지 않게 제공하는 것이 매우 중요합니다. 적절한 시기를 놓치면 루머가 금방 퍼지기 때문에 불확실성을 최대한 낮추기 위해서는 전문가 집단을 위주로 한 위기 소통능력이 매우 중요합니다.
